# Development and validation of a novel prognostic model for gastric signet ring cell carcinoma based on inflammation-nutrition indicators

**DOI:** 10.3389/fnut.2026.1623570

**Published:** 2026-04-02

**Authors:** Wangyao Li, Haitao Hu, Peng Wang, Xinxin Shao, Yujuan Jiang, Yiming Lu, Haikuo Wang, Yantao Tian

**Affiliations:** 1 Department of Pancreatic and Gastric Surgery, National Cancer Center/National Clinical Research Center for Cancer/Cancer Hospital, Chinese Academy of Medical Sciences and Peking Union Medical College, Beijing, China; 2 Department of General Surgery, China-Japan Friendship Hospital, Beijing, China

**Keywords:** gastric signet-ring cell carcinoma, inflammatory markers, nomogram, nutritional status, prognostic model

## Abstract

**Background:**

Gastric signet-ring cell carcinoma (GSRCC) is a highly aggressive subtype of gastric cancer with poor prognosis. Existing prognostic models lack integrated analysis of systemic inflammation and nutritional status. This study aimed to develop and validate a prognostic model incorporating inflammatory and nutritional indicators to enhance individualized clinical management in GSRCC patients.

**Methods:**

A total of 604 GSRCC patients who underwent curative surgery between 2014 and 2018 were retrospectively analyzed and divided into training (*n* = 368) and validation (*n* = 236) cohorts. Prognostic factors were selected using univariate analysis, multivariate Cox regression, and least absolute shrinkage and selection operator (LASSO) regression. A nomogram integrating clinicopathological features and inflammation-nutrition indicators was constructed and evaluated using calibration curves, time-dependent ROC (t-ROC) analysis, and decision curve analysis (DCA).

**Results:**

X-tile analysis determined optimal cut-off values for inflammation and nutrition markers. Multivariate analysis identified age ≥60 years, upper tumor location, T4 stage, N2–N3 stage, lymphocyte-to-CRP ratio (LCR) ≥ 8,785.7, and geriatric nutritional risk index (GNRI) ≥ 112.9 as independent predictors of overall survival (all *p* < 0.05). The combined LCR_GNRI score showed strong prognostic discrimination (*p* < 0.001). The nomogram achieved a concordance index (C-index) of 0.831, with good calibration and substantial clinical benefit confirmed by DCA.

**Conclusions:**

This study developed and validated a novel GSRCC prognostic nomogram integrating inflammatory and nutritional indicators, offering improved discrimination and clinical utility. It provides a practical tool for precise risk stratification and personalized management of GSRCC patients.

## Introduction

Gastric cancer is among the most common malignancies worldwide, ranking fifth in both incidence and mortality rate (1). Gastric signet ring cell carcinoma (GSRCC) is a distinct histological subtype, accounting for approximately 30% of all gastric cancer cases. Predominantly affecting younger individuals and females, GSRCC is characterized by unique biological features, including aggressive progression, frequent metastasis, and resistance to conventional therapies. As a result, GSRCC is associated with a significantly worse prognosis, with reported 5-year survival rates ranging from 18.4% to 44.1% (2, 3).

Inflammation has been recognized as the 7th hallmark of cancer and plays significant roles in tumor progression and metastasis. Tumor cells can trigger systemic inflammatory responses, which in turn promote cancer cell proliferation, invasion, and dissemination, while also facilitating angiogenesis (4–7). In recent years, peripheral blood inflammatory biomarkers have been widely utilized to predict prognosis across diverse cancers. Among these, neutrophil-to-lymphocyte ratio (NLR), platelet-to-lymphocyte ratio (PLR), lymphocyte-to-monocyte ratio (LMR), and lymphocyte-to-C-reactive protein ratio (LCR) have been validated as potential prognostic indicators in gastric cancer (8–11). However, relying on a single inflammatory marker may be inadequate to fully capture the complex and multifactorial prognostic landscape of patients with GSRCC.

In addition to inflammation, nutritional status plays a critical role in determining cancer prognosis. Malnutrition not only contributes to weight loss but also increases risk of infection, mortality and systemic inflammation, thereby accelerating tumor progression (12–14). Previous studies have demonstrated that nutritional prognostic scores possess strong predictive value in a variety of solid tumors, including gastric, esophageal, pancreatic, lung, and renal cancers (15–19). Given that single factor often fail to capture the dynamic interplay between inflammation and nutrition, integrated scoring systems combining both parameters have been developed. For example, the modified Glasgow Prognostic Score (mGPS) incorporates serum albumin and C-reactive protein (CRP) levels, while Naples Prognostic Score (NPS) is derived from serum albumin, total cholesterol, LMR, and NLR (20, 21). These combined indices enhance prognostic accuracy and offer more reliable tools for guiding clinical decision-making.

However, unlike conventional gastric adenocarcinoma, GSRCC predominantly affects younger patients, whose nutritional status is often underestimated or overlooked in clinical practice. Despite the well-recognized interplay among inflammation, nutrition and prognosis, no validated scoring system currently exists that specifically integrates these factors for patients with GSRCC. In this study, we aimed to develop and validate a novel integrated inflammatory-nutritional prognostic scoring system to improve the accuracy of survival prediction in GSRCC.

## Materials and methods

### Research design and participants

**Figure 1:**
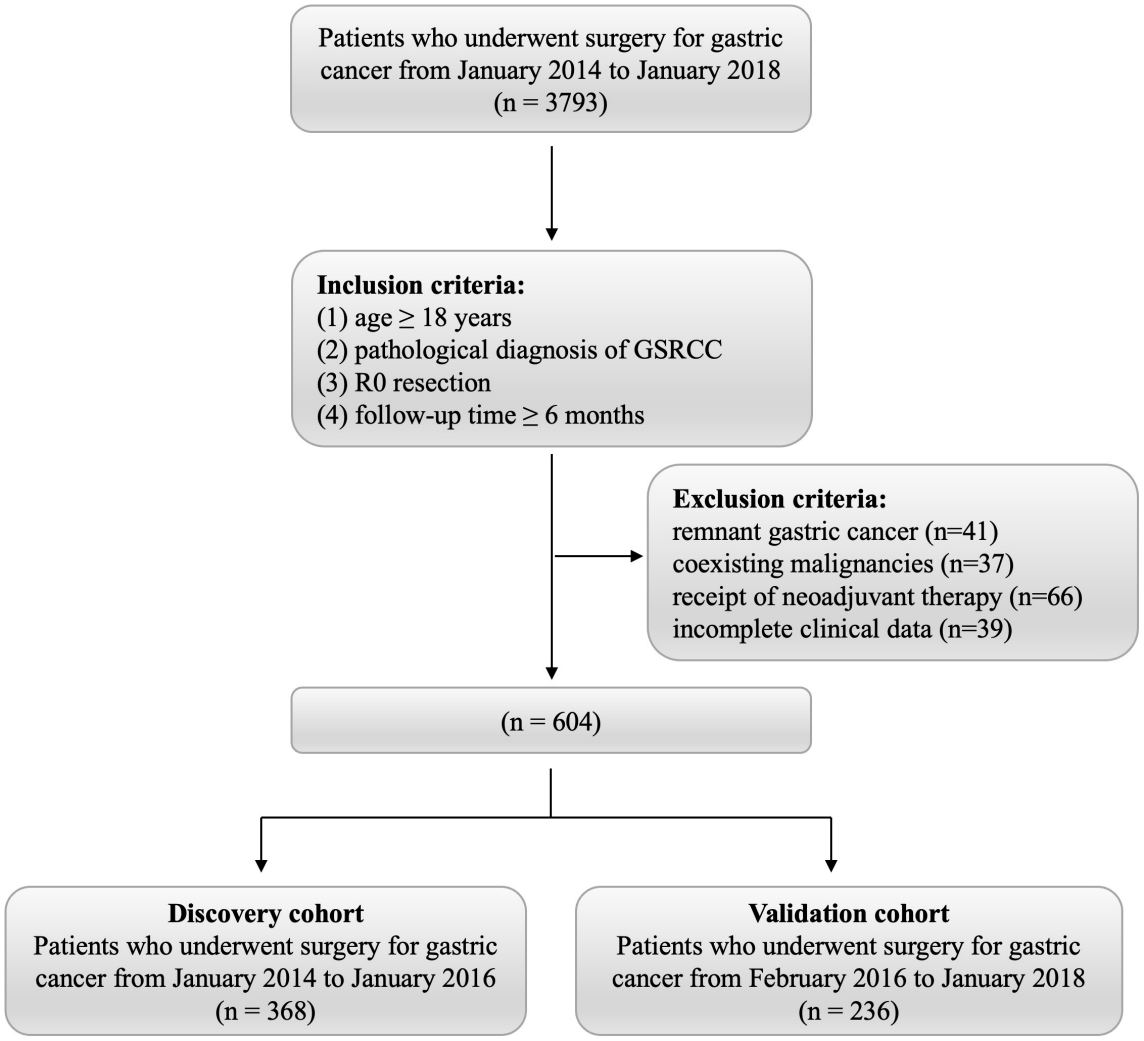
Flowchart of patient selection.

A total of 604 patients with GSRCC who received curative surgery at National Cancer Center/Cancer Hospital, Chinese Academy of Medical Sciences and Peking Union Medical College from January 2014 to January 2018 were retrospectively reviewed. The inclusion criteria were as follows: (1) age ≥ 18 years; (2) postoperative pathological diagnosis of signet ring cell carcinoma; (3) achievement of R0 resection; and (4) at least one postoperative follow-up lasting a minimum of 6 months. Exclusion criteria included: (1) remnant gastric cancer or coexisting malignancies; (2) incomplete clinical data, including missing inflammatory markers or key pathological parameters; (3) patients who received preoperative neoadjuvant chemotherapy or radiotherapy were excluded because systemic treatment can substantially modify inflammatory and nutritional parameters, thereby confounding the assessment of baseline host–tumor status that our model aims to capture. The flowchart of patient selection procedure is presented in Figure 1. Based on the date of operation, patients were categorized into discovery and validation cohorts, using January 2016 as the temporal cutoff. Ethical approval was obtained from the Ethics Committee of National Cancer Center/Cancer Hospital, Chinese Academy of Medical Sciences and Peking Union Medical College. Given the retrospective and observational nature of the study, the requirement for informed consent was waived.

### Data collection and variable definition

Clinical and pathological data were extracted from the hospital's electronic medical records and pathology database. Demographic variables included age, sex, smoking history and drinking history. Inflammatory markers consisted of white blood cell count, neutrophil count, lymphocyte count, monocyte count, platelet count, and CRP. Nutritional indicators included body mass index (BMI), serum albumin, total cholesterol (TC), triglycerides, high-density lipoprotein, low-density lipoprotein and alkaline phosphatase. Tumor-related variables encompassed tumor location, size, differentiation, perineural invasion, vascular invasion, American Joint Committee on Cancer (AJCC 8th) TNM classification, adjuvant chemotherapy, and overall survival (OS). According to the 2019 WHO histologic classification, a diagnosis of GSRCC was established when more than 50% of tumor cells exhibited typical signet ring cell morphology (22). Tumor size was defined as the maximum diameter of the lesion, and location was determined based on the tumor center, categorized as upper (cardia, fundus), middle, lower (antrum, pylorus) or entire stomach. Patient age was classified as < 60 years or ≥60 years. BMI (kg/m^2^) was categorized as normal (< 24), overweight (≥24 and < 28), and obese (≥28). Tumor size was stratified as < 4 cm and ≥4 cm. Adjuvant chemotherapy information was extracted from medical records. The most common regimens were SOX (S-1 plus oxaliplatin) and XELOX (capecitabine plus oxaliplatin), which include oral fluoropyrimidines. The primary outcome was overall survival (OS), defined as the interval from diagnosis to all-cause death or last follow-up. Follow-up data were collected up to May 2020.

### Calculation of prognostic scoring systems

The prognostic indicators analyzed in this study included NLR, PLR, LMR, LCR, CRP-to-albumin ratio (CAR), monocyte-to-albumin ratio (MAR), albumin-to-alkaline phosphatase ratio (AAPR), prognostic nutritional index (PNI), geriatric nutritional risk index (GNRI), mGPS, and NPS. Based on previously published studies, the calculation formulas were as follows:

NLR = Neutrophil count ( × 10^9^/L)/Lymphocyte count ( × 10^9^/L) (9)
PLR = Platelet count ( × 10^9^/L)/Lymphocyte count ( × 10^9^/L) (10)
LMR = Lymphocyte count ( × 10^9^/L)/Monocyte count ( × 10^9^/L) (11)
LCR = Lymphocyte count ( × 10^9^/L) × 10^4^/CRP (mg/L) (8)
CAR = CRP (mg/L)/Serum albumin (g/L) (23)
MAR = Monocyte count ( × 10^9^/L) × 10^4^/Serum albumin (g/L) (24)
AAPR = Serum albumin (g/L)/Alkaline phosphatase (U/L) (25)
PNI = Serum albumin (g/L) + 5 × Lymphocyte count ( × 10^9^/L) (26)
GNRI = 1.489 × Serum albumin (g/L) + 41.7 × BMI/22 (for males), and 1.489 × Serum albumin (g/L) + 41.7 × BMI/21 (for females) (16)

The mGPS was scored as follows: 0 for CRP ≤ 10 mg/L; 1 for CRP >10 mg/L with albumin ≥ 35 g/L; and 2 for CRP >10 mg/L with albumin < 35 g/L (20). NPS was calculated by serum albumin, TC level, LMR, and NLR, patients were grouped into three categories accordingly (21).

### Statistical analysis

Categorical variables were presented as numbers and percentages, and analyzed using the chi-square test or Fisher's exact test according to distribution. OS was assessed using univariate and multivariate Cox proportional hazards regression to estimate hazard ratios (HR) and corresponding 95% confidence intervals (CIs). To identify key prognostic factors among clinicopathological variables and reduce the risk of model overfitting, least absolute shrinkage and selection operator (LASSO) regression was performed. The optimal penalty parameter (λ) was determined via 10-fold cross-validation, selecting the λ value that minimized cross-validation error (λ.min). Variables with non-zero coefficients were considered significant predictors and were subsequently included in the multivariate Cox regression analysis. A nomogram for predicting 1-, 3-, and 5-year OS was constructed based on the independent prognostic factors identified in the multivariate model. Time-dependent receiver operating characteristic (t-ROC) curves were used to compare the area under the curve (AUC) across different combinations of inflammatory and nutritional indicators. Calibration curves for both the training and validation cohorts were generated through bootstrap resampling. Decision curve analysis (DCA) was performed to assess the clinical utility of the nomogram for 1-, 3-, and 5-year OS prediction. Survival curves were estimated using the Kaplan–Meier method and compared with the log-rank test. Additional subgroup analyses were conducted by including interaction terms (tumor location × covariates, age × covariates) in Cox models to explore potential effect modification. All statistical tests were two-sided, and a *p*-value < 0.05 was considered statistically significant. Optimal cut-off values for inflammatory markers were determined using X-tile software version 3.6.1. All statistical analyses were conducted using R software version 4.3.1.

## Results

### Clinicopathological characteristics of patients

A total of 604 patients with GSRCC were included in this study, comprising 368 cases in the discovery cohort and 236 cases in the validation cohort. Among the enrolled patients, 362 (59.9%) were at a younger age (< 60 years), highlighting a tendency for earlier onset in GSRCC. In terms of tumor differentiation, 505 cases (83.6%) had poorly differentiated tumors, indicating that low-grade differentiation is a predominant histological feature of GSRCC. The most common tumor location was in the lower part of the stomach (antrum and pylorus), observed in 290 cases (48.0%). Regarding pathological TNM (pTNM) staging, 226 (37.4%), 104 (17.2%) and 274 (45.4%) patients were classified at stage I, II and III, respectively. Detailed clinicopathological characteristics of the discovery and validation cohorts are summarized in Table 1. Baseline characteristics were comparable between the two cohorts, with no statistically significant differences observed (*p* > 0.05). The median follow-up time, estimated using the reverse Kaplan–Meier method, was 49 months. The average postoperative survival duration of our cohort was 45 months.

**Table 1:** Clinicopathological characteristics in patients with GSRCC. BMI, body mass index.

Patient features	All cases (*n* = 604)	Discovery cohort (*n* = 368)	Validation cohort (*n* = 236)	*p*-value
Gender				0.489
Male	394 (65.2%)	244 (66.3%)	150 (63.6%)	
Female	210 (34.8%)	124 (33.7%)	86 (36.4%)	
Age (years)				0.558
< 60	362 (59.9%)	224 (60.9%)	138 (58.5%)	
≥60	242 (40.1%)	144 (39.1%)	98 (41.5%)	
Smoking history				0.642
No	350 (57.9%)	216 (58.7%)	134 (56.8%)	
Yes	254 (42.1%)	152 (41.3%)	102 (43.2%)	
Drinking history				0.415
No	340 (56.3%)	212 (57.6%)	128 (54.2%)	
Yes	264 (43.7%)	156 (42.4%)	108 (45.8%)	
BMI (kg/m^2^)				0.732
Normal (< 24)	314 (52.0%)	194 (52.7%)	120 (50.9%)	
Overweight (24-28)	253 (41.9%)	150 (40.8%)	103 (43.6%)	
Obese (>28)	37 (6.1%)	24 (6.5%)	13 (5.5%)	
Tumor size (cm)				0.143
< 4	300 (49.7%)	174 (47.3%)	126 (53.4%)	
≥4	304 (50.3%)	194 (52.7%)	110 (46.6%)	
Tumor location				0.189
Upper	144 (23.8%)	88 (23.9%)	56 (23.7%)	
Middle	148 (24.5%)	92 (25.0%)	56 (23.7%)	
Lower	290 (48.0%)	170 (46.2%)	120 (50.9%)	
Entire stomach	22 (3.6%)	18 (4.9%)	4 (1.7%)	
Tumor differentiation				0.138
Poorly differentiated	505 (83.6%)	301 (81.8%)	204 (86.4%)	
Moderately differentiated	94 (15.6%)	65 (17.7%)	29 (12.3%)	
Highly differentiated	5 (0.8%)	2 (0.5%)	3 (1.3%)	
pT				0.911
T1	216 (35.7%)	134 (36.4%)	82 (34.8%)	
T2	70 (11.6%)	40 (10.9%)	30 (12.7%)	
T3	102 (16.9%)	62 (16.8%)	40 (16.9%)	
T4	216 (35.8%)	132 (35.9%)	84 (35.6%)	
pN				0.682
N0	256 (42.4%)	150 (40.8%)	106 (44.9%)	
N1	62 (10.3%)	40 (10.9%)	22 (9.3%)	
N2	110 (18.2%)	66 (17.9%)	44 (18.6%)	
N3	176 (29.1%)	112 (30.4%)	64 (27.1%)	
pTNM stage				0.459
I	226 (37.4%)	138 (37.5%)	88 (37.3%)	
II	104 (17.2%)	58 (15.8%)	46 (19.5%)	
III	274 (45.4%)	172 (46.7%)	102 (43.2%)	
Vascular invasion				0.609
No	366 (60.6%)	220 (59.8%)	146 (61.9%)	
Yes	238 (39.4%)	148 (40.2%)	90 (38.1%)	
Perineural invasion				0.337
No	286 (47.4%)	180 (48.9%)	106 (44.9%)	
Yes	318 (52.6%)	188 (51.1%)	130 (55.1%)	
Adjuvant chemotherapy				0.599
No	248 (41.0%)	148 (40.2%)	100 (42.4%)	
Yes	356 (59.0%)	220 (59.8%)	136 (57.6%)	

### Biomarkers of patients

Optimal cut-off values for inflammatory and nutritional indicators in the overall cohort were determined using X-tile analysis (Supplementary Figure S1), as follows: NLR = 1.5, PLR = 130.6, LMR = 5.7, MAR = 6.3, AAPR = 6.3, LCR = 8,785.7, PNI = 55.8, GNRI = 112.9, CAR = 0.025. Stratification of patients based on these cut-off values revealed no significant differences between the discovery and validation cohorts in any of the inflammatory or nutritional indicators (*p* > 0.05), confirming the consistency of biomarker characteristics across the study population (Table 2). Patients were stratified by GNRI using the predefined cutoff of 112.9 (GNRI < 112.9 vs. ≥112.9). As shown in Supplementary Table S1, GNRI category was significantly associated with several clinicopathological characteristics. Patients with GNRI < 112.9 were more likely to be older (≥60 years), have larger tumors (≥4 cm), and present with more advanced pT stage (*p* = 0.001). Tumor location also differed between GNRI groups (*p* = 0.026), with a higher proportion of upper tumors and entire-stomach involvement in the GNRI < 112.9 group. In contrast, pN stage and LCR did not differ significantly between GNRI strata.

**Table 2:** Biomarker characteristics in patients with GSRCC. TC, total cholesterol; TG, triglycerides; HDL, high-density lipoprotein; LDL, low-density lipoprotein; NLR, neutrophil-to-lymphocyte ratio; PLR, platelet-to-lymphocyte ratio; LMR, lymphocyte-to-monocyte ratio; CAR, CRP-to-albumin ratio; MAR, monocyte-to-albumin ratio; AAPR, albumin-to-alkaline phosphatase ratio; LCR, lymphocyte-to-CRP ratio; PNI, prognostic nutritional index; GNRI, geriatric nutritional risk index; mGPS, modified Glasgow prognostic score; NPS, Naples prognostic score.

Biomarkers	All cases (*n* = 604)	Discovery cohort (*n* = 368)	Validation cohort (*n* = 236)	*p*-value
TC (mmol/L)				0.955
< 5.7	538 (89.1%)	328 (89.1%)	210 (89.0%)	
≥5.7	66 (10.9%)	40 (10.9%)	26 (11.0%)	
TG (mmol/L)				0.560
< 1.7	500 (82.8%)	302 (82.1%)	198 (83.9%)	
≥1.7	104 (17.2%)	66 (17.9%)	38 (16.1%)	
HDL (mmol/L)				0.562
< 0.9	76 (12.6%)	44 (12.0%)	32 (13.6%)	
≥0.9	528 (87.4%)	324 (88.0%)	204 (86.4%)	
LDL (mmol/L)				0.367
< 3.34	392 (64.9%)	244 (66.3%)	148 (62.7%)	
≥3.34	212 (35.1%)	124 (33.7%)	88 (37.3%)	
NLR				0.098
< 1.5	182 (30.1%)	120 (32.6%)	62 (26.3%)	
≥1.5	422 (69.9%)	248 (67.4%)	174 (73.7%)	
PLR				0.589
< 130.6	192 (31.8%)	120 (32.6%)	72 (30.5%)	
≥130.6	412 (68.2%)	248 (67.4%)	164 (69.5%)	
LMR				0.530
< 5.7	431 (71.4%)	266 (72.3%)	165 (69.9%)	
≥5.7	173 (28.6%)	102 (27.7%)	71 (30.1%)	
CAR				0.367
< 0.025	212 (35.1%)	124 (33.7%)	88 (37.3%)	
≥0.025	392 (64.9%)	244 (66.3%)	148 (62.7%)	
MAR				0.390
< 6.3	86 (14.2%)	56 (15.2%)	30 (12.7%)	
≥6.3	518 (85.8%)	312 (84.8%)	206 (87.3%)	
AAPR				0.353
< 0.8	528 (87.4%)	318 (86.4%)	210 (89.0%)	
≥0.8	76 (12.6%)	50 (13.6%)	26 (11.0%)	
LCR				0.404
< 8785.7	144 (23.8%)	92 (25.0%)	52 (22.0%)	
≥8785.7	460 (76.2%)	276 (75.0%)	184 (78.0%)	
PNI				0.186
< 55.8	398 (65.9%)	250 (68.0%)	148 (62.7%)	
≥55.8	206 (34.1%)	118 (32.0%)	88 (37.3%)	
GNRI				0.103
< 112.9	332 (55.0%)	212 (57.6%)	120 (50.85%)	
≥112.9	272 (45.0%)	156 (42.4%)	116 (49.15%)	
mGPS				0.326
0	544 (90.0%)	332 (90.2%)	212 (89.8%)	
1	27 (4.5%)	19 (5.2%)	8 (3.4%)	
2	33 (5.5%)	17 (4.6%)	16 (6.8%)	
NPS				0.190
0	140 (23.2%)	86 (23.4%)	54 (22.9%)	
1	376 (62.2%)	236 (64.1%)	140 (59.3%)	
2	88 (14.6%)	46 (12.5%)	42 (17.8%)	

### Predictive indicators selection

**Figure 2:**
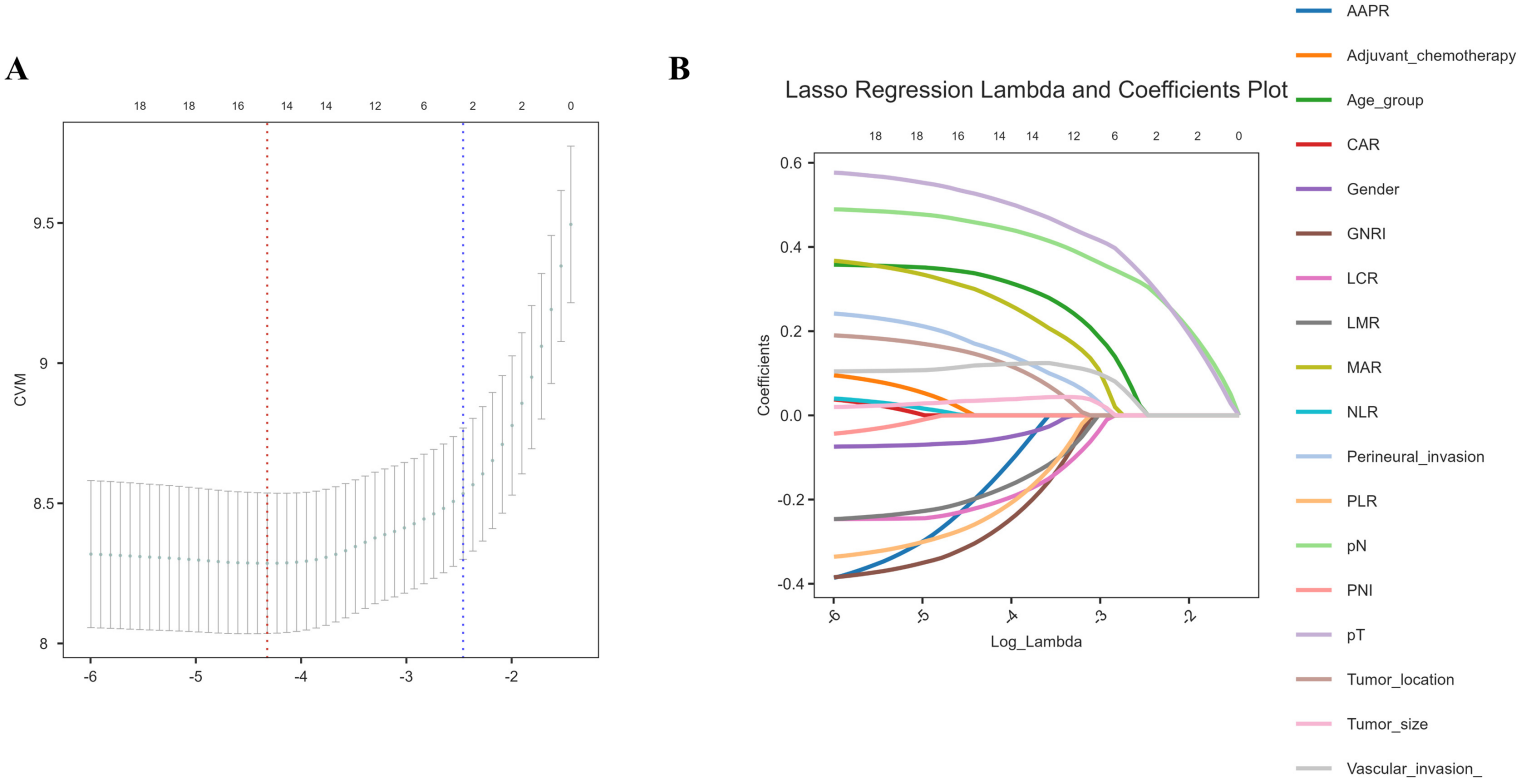
Selection of prognostic variable using LASSO regression. **(A)** Ten-fold cross-validation was used to determine the optimal λ value. The vertical dashed lines represent λ.min (left, with minimum cross-validation error) and λ.1se (right, within one standard error of the minimum); **(B)** LASSO coefficient profiles of 17 variables. Variables with non-zero coefficients were selected as potential predictors.

Univariate Cox regression analysis revealed that advanced age (≥60 years), larger tumor size (≥4 cm), upper tumor location, advanced T stage (T2–T4), advanced N stage (N2–N3), vascular invasion, perineural invasion, and receipt of adjuvant chemotherapy were significantly associated with poorer OS in the discovery cohort (*p* < 0.05). Among the inflammatory and nutritional indicators, all markers demonstrated significant associations with OS, except for serum lipid levels, mGPS and NPS (Table 3). To reduce potential multicollinearity, variables with *p* < 0.1 in univariate analysis were subjected to LASSO regression with 10-fold cross-validation. As shown in Figure 2, variables with non-zero coefficients were selected as key predictors. These included sex, age, tumor location, tumor size, vascular invasion, perineural invasion, T stage, N stage, PLR, LMR, MAR, AAPR, LCR, and GNRI. These selected variables were subsequently incorporated into a multivariate Cox regression model to identify independent prognostic factors. Although NLR, CAR, PNI, and adjuvant chemotherapy were significant in univariate analysis, these variables were not retained after LASSO selection because their coefficients were shrunk to zero and thus it was not included in the final multivariable Cox model. As shown in Table 3, advanced age (≥60 years), upper tumor location, T4 staging, N2–N3 lymph node involvement, low LCR (< 8,785.7) and low GNRI (< 112.9) emerged as independent prognostic indicators (*p* < 0.05), all of which were strongly associated with worse OS in patients with GSRCC.

**Table 3:** Univariate and multivariate analysis of clinicopathologic variables in relation to OS. TC, total cholesterol; TG, triglycerides; HDL, high-density lipoprotein; LDL, low-density lipoprotein; NLR, neutrophil-to-lymphocyte ratio; PLR, platelet-to-lymphocyte ratio; LMR, lymphocyte-to-monocyte ratio; CAR, CRP-to-albumin ratio; MAR, monocyte-to-albumin ratio; AAPR, albumin-to-alkaline phosphatase ratio; LCR, lymphocyte-to-CRP ratio; PNI, prognostic nutritional index; GNRI, geriatric nutritional risk index; mGPS, modified Glasgow prognostic score; NPS, Naples prognostic score; HR, hazard ratios; CI, confidence intervals; OS, overall survival.

Variables	Univariate analysis			Multivariate analysis		
	HR	95% CI	*p*-value	HR	95% CI	*p*-value
Gender						
Male	Reference			Reference		
Female	1.601	0.973–2.635	0.064	0.929	0.613–1.408	0.730
Age (years)						
< 60	Reference			Reference		
≥60	2.321	1.686–3.195	< 0.001	1.499	1.057–2.127	0.023
Tumor size (cm)						
< 4	Reference			Reference		
≥4	3.577	2.479–5.162	< 0.001	0.984	0.631–1.535	0.944
Tumor location						
Middle/Lower	Reference			Reference		
Upper	2.094	1.487–2.948	< 0.001	1.478	1.013–2.156	0.043
Entire stomach	2.036	0.989–4.19	0.054	2.155	0.990–4.690	0.053
Tumor differentiation						
Poorly	Reference					
Moderately/Highly	0.656	0.41–1.049	0.078			
pT						
T1	Reference			Reference		
T2	4.440	1.751–11.259	0.002	1.906	0.667–5.444	0.228
T3	7.717	3.492–17.056	< 0.001	1.898	0.703–5.123	0.206
T4	21.004	10.179–43.339	< 0.001	5.205	2.025–13.38	0.001
pN						
N0	Reference			Reference		
N1	2.123	0.908–4.964	0.083	1.111	0.45–2.742	0.82
N2	5.185	2.844–9.450	< 0.001	2.669	1.329–5.36	0.006
N3	12.913	7.598–21.945	< 0.001	4.614	2.44–8.725	< 0.001
Vascular invasion						
No	Reference			Reference		
Yes	3.671	2.625–5.135	< 0.001	1.097	0.732–1.645	0.653
Perineural invasion						
No	Reference			Reference		
Yes	5.687	3.725–8.684	< 0.001	1.407	0.839–2.359	0.195
Adjuvant chemotherapy						
No	Reference					
Yes	0.678	0.492–0.934	0.017			
TC (mmol/L)						
< 5.7						
≥5.7	0.783	0.452–1.356	0.382			
TG (mmol/L)						
< 1.7	Reference					
≥1.7	0.78	0.496–1.226	0.281			
HDL (mmol/L)						
< 0.9	Reference					
≥0.9	0.945	0.59–1.511	0.812			
LDL (mmol/L)						
< 3.34	Reference					
≥3.34	0.9	0.642–1.262	0.542			
NLR						
< 1.5	Reference					
≥1.5	1.826	1.237–2.696	0.002			
PLR						
< 130.6	Reference			Reference		
≥130.6	1.369	0.987–1.898	0.06	1.128	0.712–1.786	0.608
LMR						
< 5.7	Reference			Reference		
≥5.7	0.461	0.293–0.724	0.001	0.738	0.463–1.177	0.202
CAR						
< 0.025	1					
≥0.025	1.667	1.163–2.391	0.005			
MAR						
< 6.3	Reference			Reference		
≥6.3	2.251	1.248–4.058	0.007	1.486	0.766–2.883	0.242
AAPR						
< 0.8	Reference					
≥0.8	0.24	0.106–0.544	0.001	0.642	0.263–1.57	0.332
LCR						
< 8785.7	Reference			Reference		
≥8785.7	0.455	0.326–0.637	< 0.001	0.483	0.290–0.807	0.005
PNI						
< 55.8	Reference					
≥55.8	0.425	0.285–0.634	< 0.001			
GNRI						
< 112.9	Reference			Reference		
≥112.9	0.543	0.388–0.761	< 0.001	0.617	0.427–0.89	0.010
mGPS						
0	Reference					
1	1.225	0.679–2.210	0.500			
2	0.391	0.097–1.579	0.187			
NPS						
0	Reference					
1	1.486	0.862–2.564	0.154			
2	1.404	0.924–2.133	0.112			

### Predictive value of combined inflammation-nutrition indicators

To further assess the prognostic value of integrated inflammatory and nutritional markers, we combined the most predictive inflammatory marker (LCR) with the most predictive nutritional marker (GNRI) to evaluate the performance of different combinations vs. individual indicators. Three combination models were proposed:

- **LCR_GNRI1:** Patients with both LCR < 8,785.7 and GNRI < 112.9 were assigned 0 points; those with either LCR ≥ 8,785.7 or GNRI ≥ 112.9 were assigned 1 point; and those with both LCR ≥ 8,785.7 and GNRI ≥ 112.9 were assigned 2 points;
- **LCR_GNRI2:** Patients with GNRI < 112.9 were assigned 0 points; those with GNRI ≥ 112.9 and LCR < 8,785.7 received 1 point; and those with both GNRI ≥ 112.9 and LCR ≥ 8,785.7 received 2 points;
- **LCR_GNRI3:** Patients with LCR < 8,785.7 were assigned 0 points; those with LCR ≥ 8,785.7 and GNRI < 112.9 received 1 point; and those with both LCR ≥ 8,785.7 and GNRI ≥ 112.9 received 2 points.

**Figure 3:**
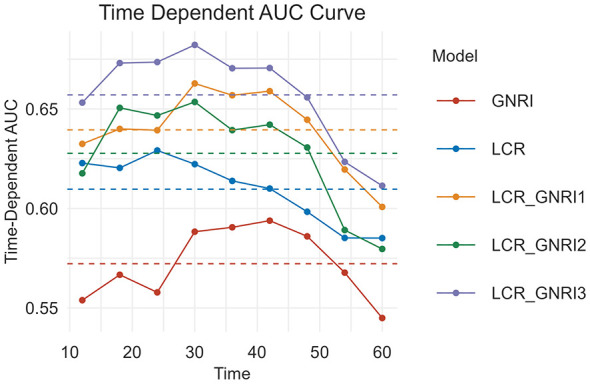
t-ROC curves of LCR, GNRI, and the three LCR_GNRI combinations (LCR_GNRI1, LCR_GNRI2, and LCR_GNRI3) for prediction of OS. The horizontal axis shows postoperative survival time, and the vertical axis displays the estimated AUC. LCR, lymphocyte-to-CRP ratio; GNRI, geriatric nutritional risk index; t-ROC, time-dependent receiver operating characteristic curve; AUC, area under the curve; OS, overall survival.

**Figure 4:**
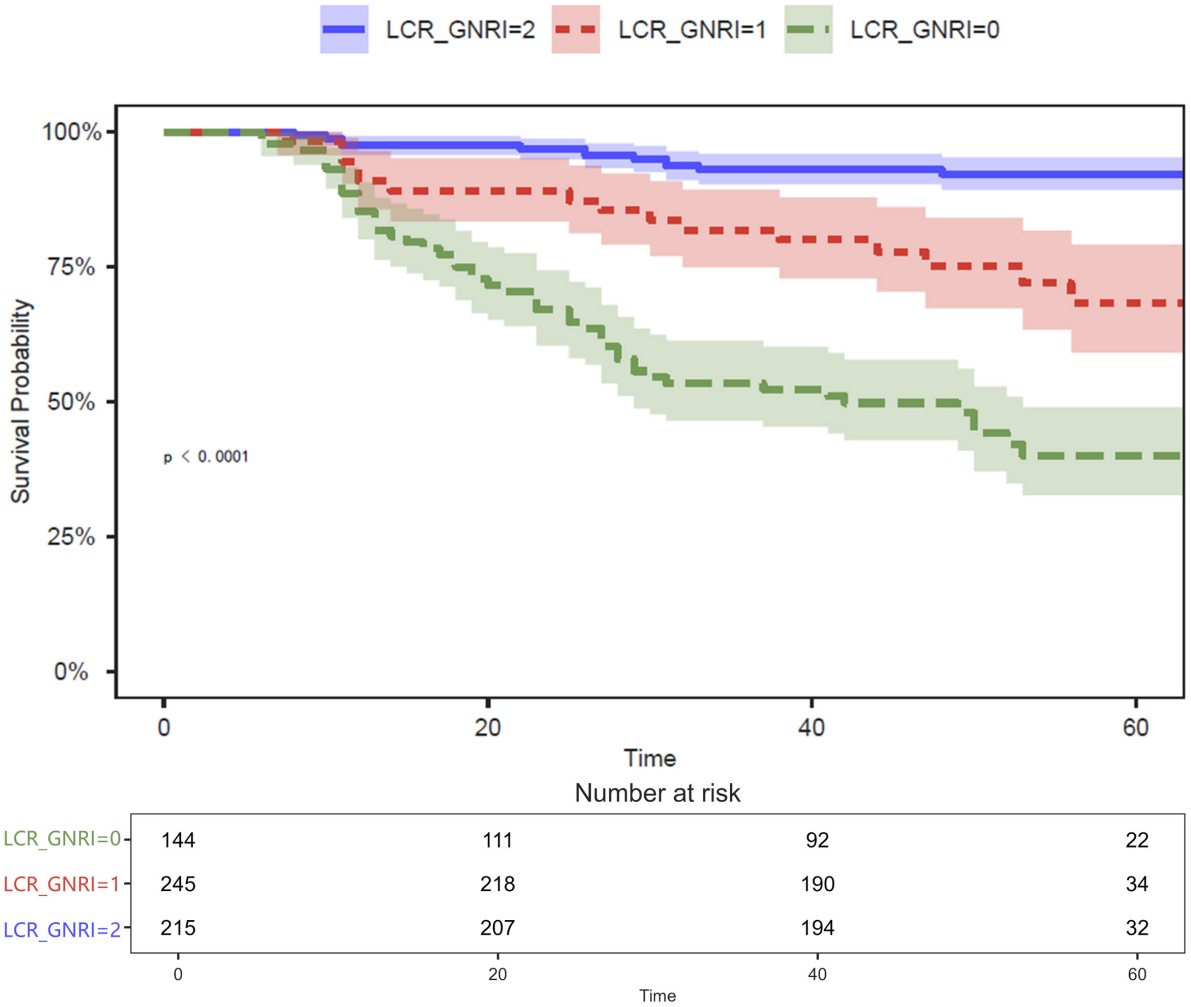
Kaplan–Meier survival curves for OS in patients with GSRCC stratified by LCR_GNRI score. Patients with a score of 2 exhibited the most favorable prognosis, followed by those with a score of 1, while those with a score of 0 had the poorest survival outcomes. Prognostic differences among the three groups were statistically significant (*p* < 0.0001). Shaded areas indicate 95% confidence intervals, and numbers at risk are shown below. OS, overall survival; GSRCC, gastric signet ring cell carcinoma.

As illustrated in Figure 3, the LCR_GNRI3 model yielded the highest AUC, indicating superior predictive performance compared to the other models. Kaplan–Meier survival analysis further confirmed significant differences in OS among the three groups defined by LCR_GNRI3 (Figure 4). Accordingly, LCR_GNRI3 was selected as the final integrated prognostic scoring system and designated as the LCR_GNRI score.

### Construction and validation of nomogram

**Figure 5:**
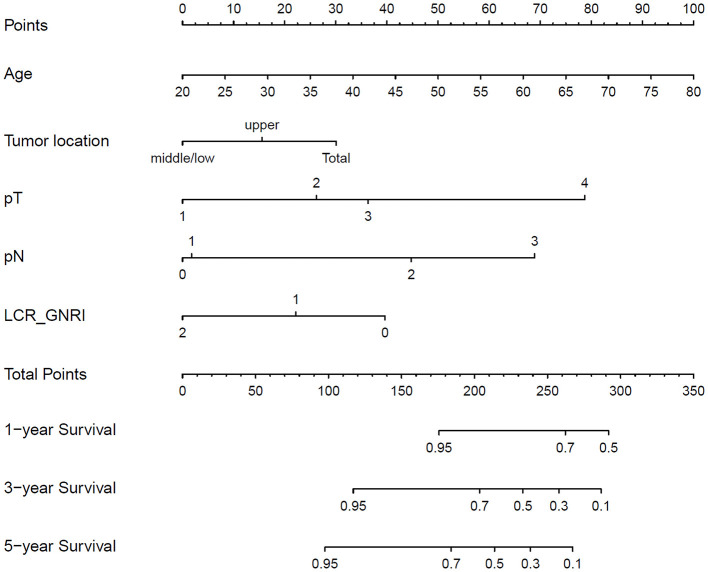
Nomogram developed based on inflammatory and nutritional indicators to predict 1-, 3-, and 5-year OS in patients with GSRCC. Variables included in the nomogram are age, tumor site, pathological T stage, pathological N stage, and LCR_GNRI score. OS, overall survival; GSRCC, gastric signet ring cell carcinoma.

**Figure 6:**
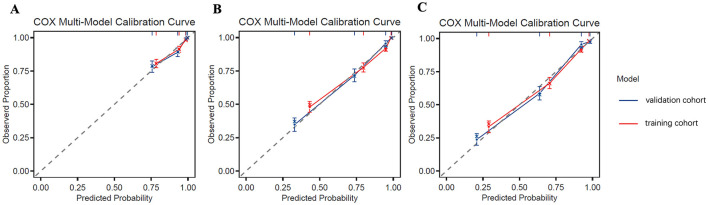
Calibration curves of the nomogram for predicting 1-year **(A)**, 3-year **(B)**, and 5-year OS **(C)** in patients with GSRCC. Red and blue lines represent predicted survival in the training and validation cohorts, respectively. Solid lines indicate predicted probabilities, while the dashed diagonal line represents ideal calibration. OS, overall survival; GSRCC, gastric signet ring cell carcinoma.

**Figure 7:**
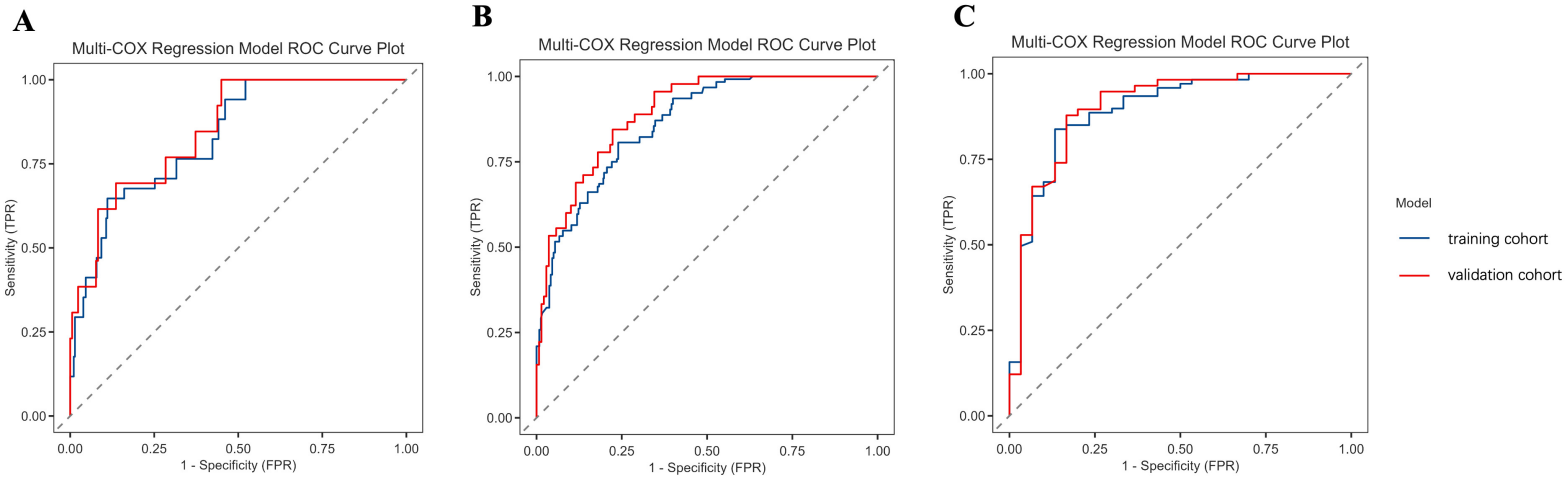
ROC curves comparing the predictive performance of the nomogram in the training and validation cohorts for 1-year **(A)**, 3-year **(B)**, and 5-year OS **(C)** in patients with GSRCC. OS, overall survival; GSRCC, gastric signet ring cell carcinoma; ROC, receiver operating characteristic curve.

**Figure 8:**
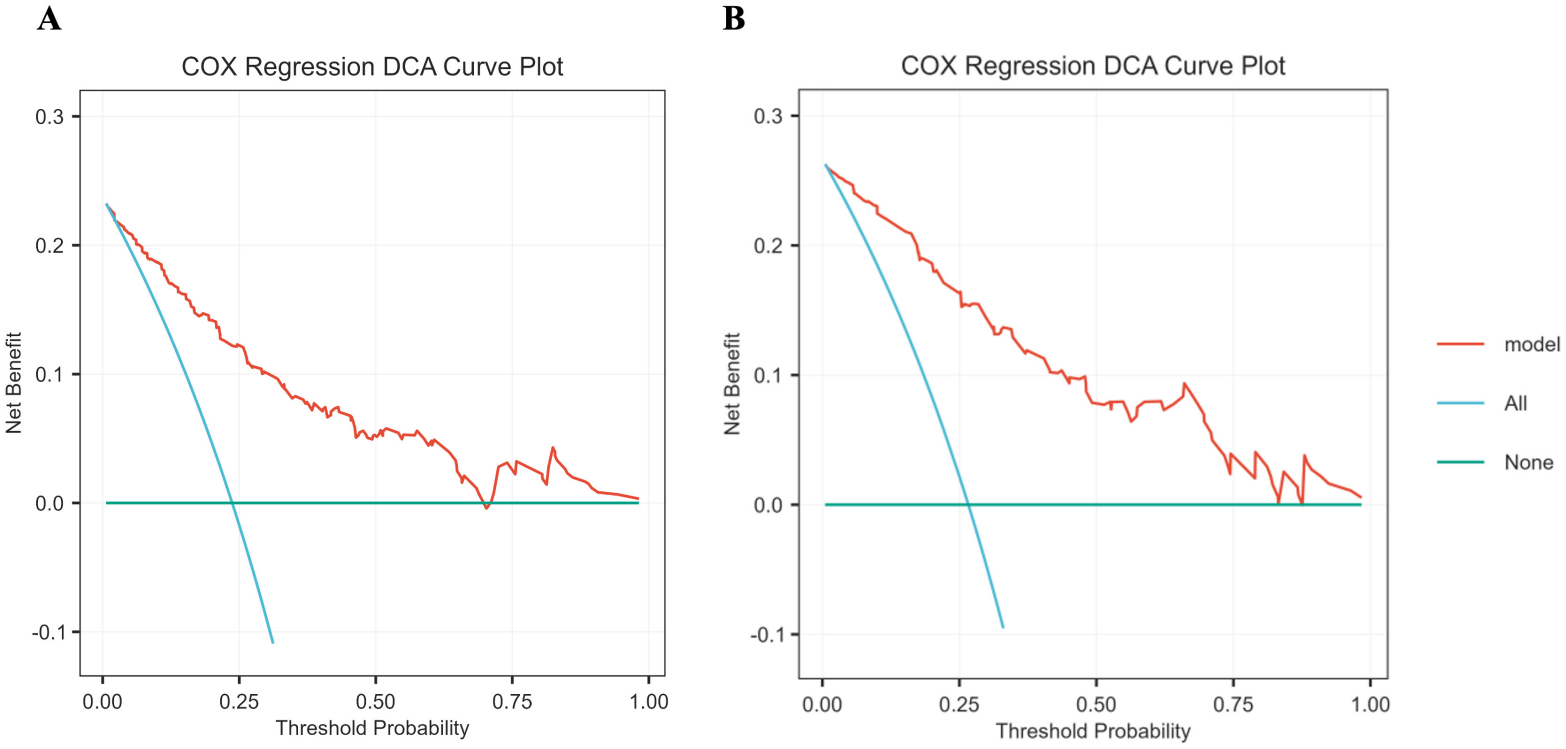
DCA comparing the clinical utility of the nomogram for predicting 5-year OS in patients with GSRCC. **(A)** Training cohort; **(B)** Validation cohort. DCA, decision curve analysis; OS, overall survival; GSRCC, gastric signet ring cell carcinoma.

Based on the identified independent prognostic factors, a nomogram was developed incorporating age, tumor site, T stage, N stage, and the LCR_GNRI score to predict OS in GSRCC patients. The nomogram demonstrated strong discriminatory power, with a concordance index (C-index) of 0.831 (Figure 5). In the discovery cohort, the AUCs for 1-, 3- and 5- year OS were 0.83, 0.862, and 0.893, respectively. Calibration curves showed excellent agreement between predicted and observed survival probabilities at 1, 3, and 5 years in both cohorts, indicating good model calibration (Figure 6). In the discovery cohort, the AUCs for 1-, 3- and 5- year OS were 0.83, 0.862, and 0.893, respectively. In the validation cohort, the corresponding AUCs were 0.85, 0.893, and 0.902 (Figure 7). In terms of clinical utility, DCA demonstrated a clear net benefit of the nomogram for predicting 5-year OS in both the discovery and validation sets (Figure 8).

To further evaluate the clinical utility of our nomogram, we compared its discriminative ability with the AJCC TNM staging system using AUC. The nomogram demonstrated a C-index of 0.831, which was higher than the C-index of the AJCC TNM staging system (C-index = 0.749). The AUC for 1-, 3-, and 5-year OS prediction was 0.837, 0.862, and 0.866 for the nomogram, respectively, compared to 0.706, 0.758, and 0.781 for the AJCC TNM system, further confirming its added clinical value over conventional staging (Supplementary Figure S2).

### Subgroup analyses and interaction testing

To further evaluate the borderline prognostic effect of “entire stomach” tumor location observed in multivariable analysis (*p* = 0.053), we performed subgroup analyses. We found that the prognostic effect of “entire stomach” tumor location was not consistent across subgroups. A statistically significant interaction was observed with sex (*p* for interaction = 0.001): the association appeared stronger in males (HR 11.309, 95% CI 4.015–31.854) but not in females. No meaningful interactions were detected with age group, tumor size, pT stage, or pN stage (all *p* for interaction > 0.05) (Supplementary Figure S3). These estimates should be interpreted cautiously because the number of patients with “entire stomach” involvement was very small in several strata (e.g., only 4 males), resulting in wide confidence intervals and unstable estimates. We then validate the model's performance across different age strata. Age-stratified subgroup analyses (< 60 vs. ≥60 years) showed consistent associations of LCR, GNRI, and LCR_GNRI with OS, with no significant interactions (*p* for interaction: 0.112, 0.429, and 0.12, respectively) (Supplementary Figure S4). We further assessed discrimination of the multivariable Cox model, which showed good performance in both age strata, with an AUC of 0.902 in patients < 60 years and 0.823 in patients ≥60 years, indicating stable predictive ability, particularly in the younger cohort (Supplementary Figure S5).

## Discussion

GSRCC is a distinct histological subtype of gastric cancer, characterized by increasing global incidence, aggressive biological behavior, and poor clinical outcomes (27). Growing evidence underscores the crucial role of systemic inflammation and nutritional status in cancer initiation, progression, and prognosis (4, 7). In this study, we conducted a retrospective study to develop and validate a prognostic model for GSRCC that integrates both inflammatory and nutritional biomarkers. Our findings revealed that the inflammatory marker LCR and the nutritional marker GNRI were both significantly associated with OS in GSRCC patients. Multivariate Cox regression analysis further identified advanced age (≥60 years), upper tumor location, T4 staging, N2–N3 lymph node involvement, low LCR (< 8,785.7) and low GNRI (< 112.9) as independent predictors of poor OS. Based on these variables, we constructed a nomogram that demonstrated robust prognostic performance, with a C-index of 0.831. Calibration curves indicated strong agreement between predicted and actual 1-, 3-, and 5-year OS, and DCA confirmed a substantial net clinical benefit. Notably, for the variable selection process using LASSO regression, we compared two commonly used penalty parameters: λ.min, which minimizes the cross-validation error, and λ.1se, which selects the model within one standard error of the minimum cross-validation error. We compared the models with λ.min and λ.1se, and found that the λ.min model demonstrated superior predictive accuracy, as evidenced by a higher concordance index and improved AUC (Supplementary Figure S6). Although λ.1se could have been used to select a simpler model with fewer variables, we found that it resulted in a higher cross-validation error and reduced predictive performance. Therefore, we believe that λ.min was the most appropriate choice for this study to ensure a robust and accurate prognostic model. Taken together, these results support the clinical utility of our model as a statistically sound and practically meaningful prognostic tool. It may facilitate individualized risk stratification and assist clinicians in formulating more tailored management strategies for patients with GSRCC.

Our study first integrates inflammatory and nutritional markers for prognostic prediction in GSRCC patients. Conventional prognostic models predominantly rely on clinicopathological parameters such as TNM stage and tumor size (28, 29). In contrast, our study incorporates LCR and GNRI, providing a more comprehensive and biologically informed approach to risk stratification. LCR, as a novel inflammatory marker, reflects the balance between host immune competence and systemic inflammation by combining lymphocyte count and CRP levels (8). Lymphocytes are essential mediators of antitumor immunity, limiting tumor progression through cytokine production and cytotoxic killing (30). Accordingly, tumor-infiltrating lymphocytes reflect the strength of the local cytotoxic immune response (31). In contrast, lymphopenia indicates impaired host immune competence and is associated with worse oncological outcomes (32). CRP, an acute-phase reactant, is frequently elevated in the presence of tumor-promoting inflammation (33). Cells activated by monomeric CRP can stimulate intracellular signaling pathways, including the activation of the NF-kB transcription factor (34). CRP directly binds fibronectin stimulating the retention of monocytes in the TIME (tumor immune microenvironment) (35). Moreover, CRP can enhance cytotoxic T lymphocyte-mediated cell lysis and, on the other hand, can promote a sustained pro-inflammatory and pro-tumor immune microenvironment (34). Tumor progression and metastasis result from interactions between tumor cells and TIME. Therefore, LCR may reflect the antitumor cytotoxicity of the intertumoral immune response in TIME and affect tumor. Regarding gastric cancer, a particular focus has been placed on the LCR. Several studies have shown that LCR outperforms other inflammation-based scores, such as CAR, NLR, LMR, and PLR, in predicting overall survival in gastric cancer (36, 37). Notably, a multicenter study comparing the predictive performance of 16 systemic inflammatory indices identified LCR as the strongest marker of systemic inflammatory burden (38). In our study, patients with LCR < 8,785.7 had significantly worse overall survival, likely due to the combined impact of systemic inflammation and impaired immune surveillance.

GNRI, derived from serum albumin and BMI, reflects nutritional reserve and chronic catabolic status. Malnutrition can impair immune function, reduce tolerance to anticancer treatment, and potentially shape an unfavorable tumor microenvironment (39). Recent studies have consistently demonstrated its prognostic value in various malignancies (15–19). Malnutrition not only reduces treatment tolerance but may also facilitate tumor progression by weakening host immune defenses (40). In line with previous findings (16), we observed that a GNRI < 112.9 was significantly associated with poorer survival in GSRCC patients. Notably, GNRI was also associated with several clinicopathological features in our cohort, including age, tumor size, pT stage, and tumor location, suggesting that nutritional compromise may partially reflect baseline disease burden and anatomic factors (Supplementary Table S1), consistent with previous reports (41, 42). Importantly, GNRI showed no significant association with LCR, indicating that GNRI captures a nutritional dimension that is not redundant with systemic inflammation and therefore provides complementary prognostic information. The integration of LCR and GNRI highlights their potential to reflect distinct yet complementary biological processes influencing cancer progression. Inflammation may promote tumor aggressiveness through mechanisms such as NF-κB pathway activation, while malnutrition may compromise antitumor immunity (16). The combined use of inflammation- and nutrition-based markers strengthens the biological rationale of our model and may more accurately reflect the overall health status and outcome risk in patients with GSRCC. These findings provide a foundation for future research and hold promise for broader clinical application.

Using X-tile analysis, we identified optimal cutoffs of 8,785.7 for LCR and 112.9 for GNRI. Published studies in gastrointestinal cancers generally report an LCR cutoff between 6,300 and 12,000 (43–45), and our threshold lies within this interval, supporting the robustness of our findings. Okugawa et al. (43) evaluated 551 patients with gastric cancer undergoing curative surgery and applied an LCR cutoff of 8,350, which is close to our value. The variability in reported LCR cutoffs likely reflects differences in patient case mix, perioperative management, treatment strategies, and sample size. For GNRI, our optimal cutoff of 112.9 is higher than the thresholds of 104 and 98 reported by Tanabe et al. (47) and Ide et al. (46), respectively. This difference may relate to the distinctive characteristics of our gastric signet-ring cell carcinoma cohort, abbreviated as GSRCC. Nearly 60% of our patients were younger than 65 years, and the cohort also had relatively high serum albumin levels, with a mean of 44 g/L, which may reflect the benefits of nutritional management and contribute to higher GNRI values. These cutoffs may therefore provide more sensitive prognostic stratification in GSRCC. Nevertheless, they should be validated in larger independent cohorts, and future studies should evaluate their generalizability across gastric cancer subtypes and diverse populations.

Age was reaffirmed in this study as a critical prognostic factor in GSRCC. Our results demonstrated that patients aged ≥60 years had significantly worse survival outcomes, consistent with findings from previous studies (48). Older individuals often present with comorbidities such as cardiovascular disease or diabetes, which may exacerbate disease burden, reduce tolerance to anti-cancer therapies, and adversely affect overall prognosis (49). Moreover, age-related immune senescence may impair antitumor immune surveillance, thereby facilitating tumor progression (50). Although GSRCC tends to occur more frequently in younger individuals, our data underscore that advanced age remains a non-negligible determinant of poor prognosis in this patient population. Another noteworthy finding is the prognostic significance of tumor location. Patients with tumors located in the upper stomach exhibited significantly worse survival compared to those with tumors in other gastric regions. This disparity may be attributed to distinct anatomical and biological characteristics associated with upper gastric tumors (51). Tumors in this region often involve the gastroesophageal junction (GEJ) and lie in close proximity to the diaphragm, making them more prone to mediastinal lymph node metastasis (42). Notably, we also observed an association between nutritional status and tumor location: GNRI categories differed significantly across tumor locations (Supplementary Table S1, *p* = 0.026), with a higher proportion of upper and entire-stomach tumors in the low-GNRI group, supporting the possibility of greater nutritional compromise with proximal/GEJ involvement. These findings suggest that patients with upper gastric or GEJ involvement may benefit from more intensive preoperative evaluation and tailored multimodal treatment strategies to improve survival outcomes. Although “entire stomach” tumor location showed borderline significance in multivariable analysis, our subgroup analysis suggested potential heterogeneity by sex (Supplementary Figure S3). However, the number of patients with “entire stomach” involvement was very limited, leading to imprecise estimates and occasional non-estimable effects in some strata. Therefore, we did not include this variable in the final nomogram to reduce the risk of overfitting and to maintain model stability. Further validation in larger, multicenter cohorts is warranted to clarify its prognostic value.

Our nomogram demonstrated superior discriminative performance, with a C-index of 0.831, substantially higher than those reported in previous prognostic models for GSRCC. For example, Shao et al. (52) reported a C-index of 0.76 in a SEER-based model, while Wang et al. (25) achieved 0.78 in a multicenter cohort. Additionally, DCA confirmed a meaningful net clinical benefit across multiple time points, with particularly strong utility in predicting 5-year OS. The combination of robust discriminatory ability and practical utility positions our model as a promising tool for prognostic evaluation in GSRCC (53). Despite these strengths, several limitations should be acknowledged. First, the retrospective nature of this study may introduce selection bias. Although strict inclusion and exclusion criteria were employed to mitigate this risk, the inherent constraints of retrospective data remain. The training and validation cohorts were separated by surgical date, using January 2016 as a temporal cutoff, to perform temporal validation. This design evaluates whether a model developed from earlier patients remains applicable to patients treated in a later period. However, advances in treatment over time may introduce a cohort effect, as improvements in surgical techniques, adjuvant therapies, and postoperative care could contribute to better outcomes in the later cohort. Second, the study was conducted at a single center, which may affect the generalizability of the findings to broader populations. Although our cohort shares similar characteristics, such as age, gender, and tumor location, with those reported in multicenter or population-based studies, these results should be interpreted with caution (54–56). To enhance the external validity of our model, we propose that future studies be conducted across multiple centers, incorporating diverse patient populations from different geographic locations. Third, although receipt of adjuvant chemotherapy was recorded, detailed treatment exposure (e.g., cycles, dose intensity, and treatment modifications) was not uniformly available, and adjuvant chemotherapy was not retained in the final prognostic model after LASSO selection. Previous studies have suggested that reductions in inflammation during therapy may be associated with improved survival outcomes (57). Meanwhile, as neoadjuvant chemotherapy–associated lymphopenia may alter the LCR_GNRI score, thereby identifying a high-risk subgroup, it seems prudent to include neoadjuvant therapy. Therefore, future studies should incorporate treatment-related factors to provide a more comprehensive assessment of prognosis in patients with GSRCC, as these variables may modify the associations between inflammation, nutrition and survival. Fourth, this study was not designed to explore underlying biological mechanisms. Although our findings support the prognostic value of inflammation–nutrition markers, the mechanistic links between systemic inflammation, nutritional status, and tumor behavior in GSRCC remain to be clarified. Future basic research should investigate immune-cell modulation and cytokine signaling in the tumor microenvironment, as well as how nutritional deficits influence tumor progression, metastasis, and treatment response. Additionally, this study did not evaluate the prognostic significance of dynamic changes in inflammatory and nutritional markers over time. Serial monitoring of these markers may offer incremental prognostic value and should be investigated in future prospective studies to clarify how they vary with disease progression and treatment response. Future prospective, multicenter studies are warranted to validate the accuracy and applicability of this model across diverse populations.

Refinement of the model may offer new avenues for individualized treatment strategies in patients with GSRCC. Based on our findings, we propose that patients with LCR_GNRI score of 0 or 1 should be considered for more aggressive treatment approaches, including neoadjuvant chemotherapy and closer postoperative monitoring. These patients have a higher risk of poor survival outcomes, and personalized management strategies could improve their prognosis. On the other hand, patients classified as low-risk (LCR_GNRI scores of 2) could be managed with standard clinical protocols and less intensive monitoring, in line with current clinical guidelines. This risk-based approach would allow for more tailored treatment, improving the overall management of GSRCC patients.

## Conclusions

Our research integrated inflammation and nutritional indicators to develop and validate a GSRCC prognostic nomogram, outperforming existing models in discrimination and clinical utility. It offers a novel tool for precise prognostic evaluation and personalized treatment, particularly for identifying and managing high-risk GSRCC patients.

## Data Availability

The raw data supporting the conclusions of this article will be made available by the authors, without undue reservation.
