# Supplementary material for: Development and validation of a novel prognostic model for gastric signet ring cell carcinoma based on inflammation-nutrition indicators
Source: Front Nutr. 2026 Apr 2;13:1623570. doi: 10.3389/fnut.2026.1623570 (PMC13084756; doi:10.3389/fnut.2026.1623570)
Supplement: Supplementary file 1 [file Table_1.docx]

Supplementary Material

**Table S1**. Clinicopathological characteristics of patients with GSRCC stratified by GNRI category (<112.9 vs ≥112.9). GSRCC, gastric signet ring cell carcinoma; LCR, lymphocyte-to-C-reactive protein ratio; GNRI, Geriatric Nutritional Risk Index.

| **Variable** | **All cases**  **(n=604)** | **GNRI<112.9**  **(n=332)** | **GNRI≥112.9**  **(n=272)** | ***p*-value** |
| --- | --- | --- | --- | --- |
| **Gender** |  |  |  | 0.031 |
| Male | 394 (65.2%) | 204 (61.5%) | 190 (69.9%) |  |
| Female | 210 (34.8%) | 128 (38.6%) | 82 (30.2%) |  |
| **Age (years)** |  |  |  | <0.001 |
| <60 | 362 (59.9%) | 176 (53.0%) | 186 (68.4%) |  |
| ≥60 | 242 (40.1%) | 156 (47.0%) | 86 (31.6%) |  |
| **Tumor size（cm）** |  |  |  | 0.002 |
| <4 | 300 (49.7%) | 146 (44.0%) | 154 (56.6%) |  |
| ≥4 | 304 (50.3%) | 186 (56.0%) | 118 (43.4%) |  |
| **Tumor location** |  |  |  | 0.026 |
| Middle/Lower | 438 (72.5%) | 227 (68.4%) | 211 (77.6%) |  |
| Upper | 144 (23.8%) | 89 (26.8%) | 55 (20.2%) |  |
| Entire stomach | 22 (3.6%) | 16 (4.8%) | 6 (2.2%) |  |
| **pT** |  |  |  | 0.001 |
| T1 | 216 (35.7%) | 98 (29.5%) | 118 (43.4%) |  |
| T2 | 70 (11.6%) | 36 (10.8%) | 34 (12.5%) |  |
| T3 | 102 (16.9%) | 60 (18.1%) | 42 (15.4%) |  |
| T4 | 216 (35.8%) | 138 (41.6%) | 78 (28.7%) |  |
| **pN** |  |  |  | 0.216 |
| N0 | 256 (42.4%) | 128 (38.6%) | 128 (47.1%) |  |
| N1 | 62 (10.3%) | 36 (10.8%) | 26 (9.6%) |  |
| N2 | 110 (18.2%) | 64 (19.3%) | 46 (16.9%) |  |
| N3 | 176 (29.1%) | 104 (31.3%) | 72 (26.5%) |  |
| **LCR** |  |  |  | 0.132 |
| <8785.7 | 144 (23.8%) | 87 (26.2%) | 57 (21.0%) |  |
| ≥8785.7 | 460 (76.2%) | 245 (73.8%) | 215 (79.0%) |  |
