# Supplementary material for: Development and validation of a novel prognostic model for gastric signet ring cell carcinoma based on inflammation-nutrition indicators
Source: Front Nutr. 2026 Apr 2;13:1623570. doi: 10.3389/fnut.2026.1623570 (PMC13084756; doi:10.3389/fnut.2026.1623570)
Supplement: Supplementary file 2 [file Image_1.pdf]

Supplementary Material

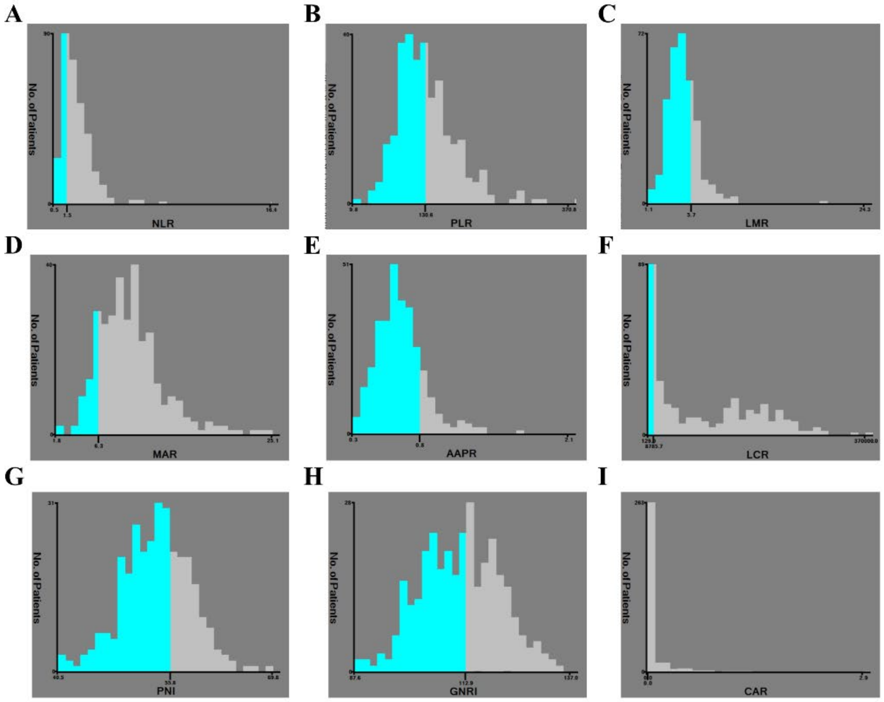

Figure S1. X-tile analysis was used to identify the optimal thresholds for NLR (A), PLR (B), LMR (C), MAR (D), AAPR (E), LCR (F), PNI (G), GNRI (H), and CAR (I).

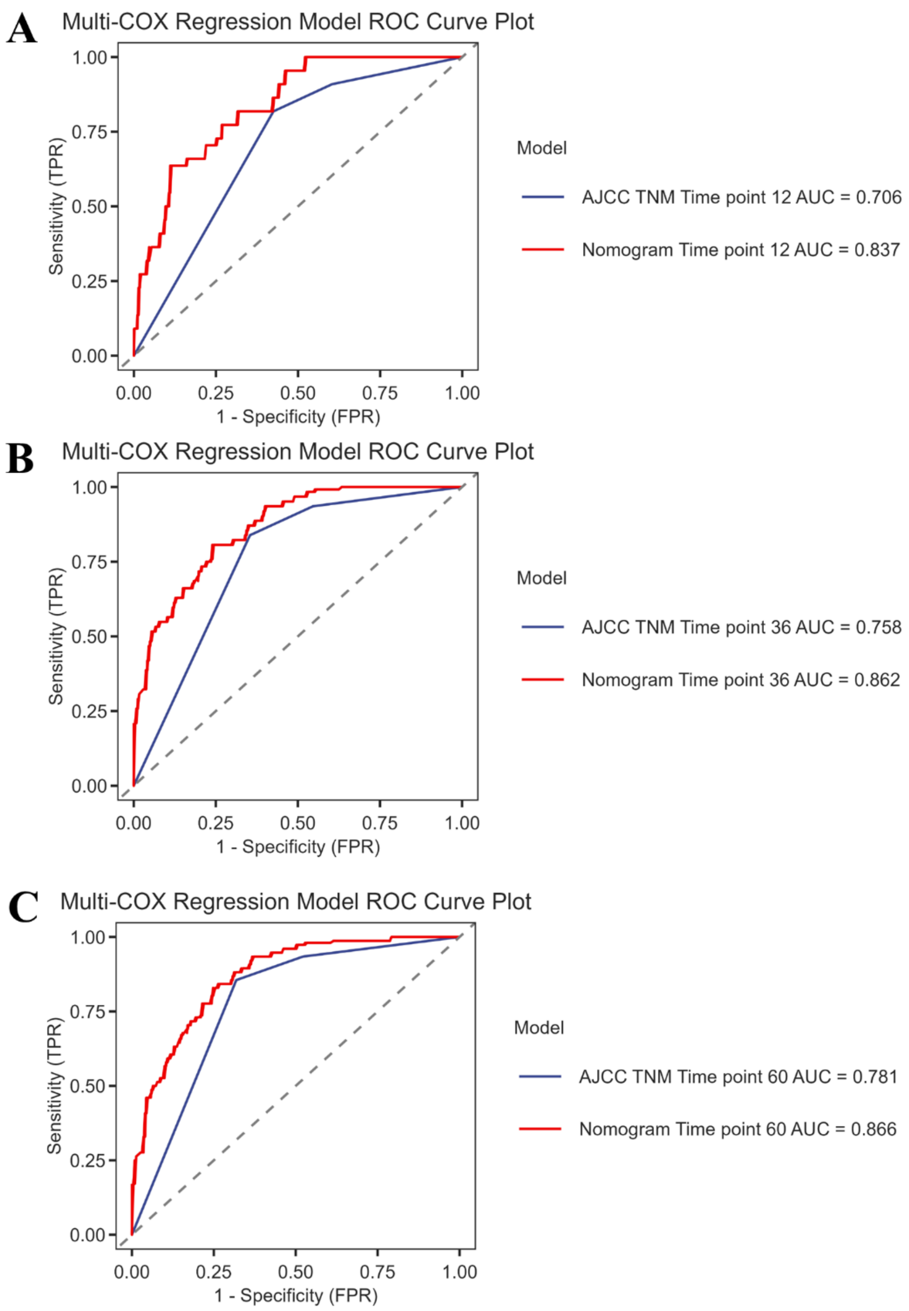

Figure S2. ROC curves comparing the predictive performance of the nomogram and AJCC TNM staging system for 1-year (A), 3-year (B), and 5-year OS (C) in patients with GSRCC. OS, overall survival; GSRCC, gastric signet ring cell carcinoma; ROC, receiver operating characteristic curve.

Subgroup Interaction Regression Forest Plot

Variable	Group	Count	Percent	HR(95%CI)	P for Interaction
**Gender**	**Tumor_location**				**0.001**
Female					
	Middle/Lower	159	89.831	ref	
	Entire stomach	18	10.169	1.079(0.382,3.048)	
Male					
	Middle/Lower	279	98.587	ref	
	Entire stomach	4	1.413	11.309(4.015,31.854)	
**Age**	**Tumor_location**				**0.394**
<60					
	Middle/Lower	283	95.932	ref	
	Entire stomach	12	4.068	2.58(0.926,7.186)	
≥60					
	Middle/Lower	155	93.939	ref	
	Entire stomach	10	6.061	1.295(0.466,3.601)	
**Tumor size**	**Tumor_location**				**0.612**
<4cm					
	Middle/Lower	241	95.257	ref	
	Entire stomach	12	4.743	1.573(0.374,6.618)	
≥4cm					
	Middle/Lower	197	95.169	ref	
	Entire stomach	10	4.831	2.297(0.993,5.314)	
**pT**	**Tumor_location**				**0.995**
T1−2					
	Middle/Lower	238	96.748	ref	
	Entire stomach	8	3.252	Inf(Inf,Inf)	
T3−4					
	Middle/Lower	200	93.458	ref	
	Entire stomach	14	6.542	2.022(0.975,4.196)	
**pN**	**Tumor_location**				**0.993**
N0−1					
	Middle/Lower	246	96.094	ref	
	Entire stomach	10	3.906	Inf(Inf,Inf)	
N2−3					
	Middle/Lower	192	94.118	ref	
	Entire stomach	12	5.882	2.416(1.164,5.014)	

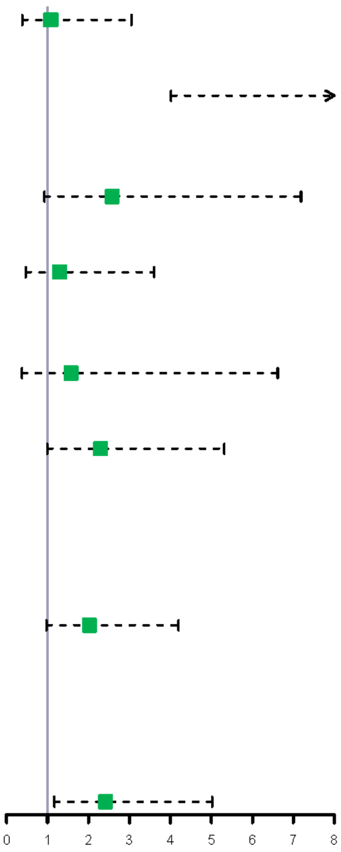

Figure S3. Subgroup interaction analysis evaluating the association between tumor location and OS in patients with GSRCC, stratified by sex, age, tumor size, pT stage, and pN stage. OS, overall survival; GSRCC, gastric signet ring cell carcinoma.

Subgroup Interaction Regression Forest Plot

Variable	Group	Count	Percent	HR(95%CI)	P for Interaction
**Age**	**LCR**				**0.112**
<60					
	<8785.7	65	17.956	ref	
	≥8785.7	297	32.645	0.379(0.224,0.638)	
≥60					
	<8785.7	79	67.355	ref	
	≥8785.7	163	51.381	0.702(0.456,1.081)	
**Age**	**GNRI**				**0.429**
<60					
	<112.9	176	64.463	ref	
	≥112.9	186	35.537	0.516(0.312,0.852)	
≥60					
	<112.9	156	17.956	ref	
	≥112.9	86	39.503	0.708(0.447,1.12)	
**Age**	**LCR_GNRI**				**0.12**
<60					
	0	65	82.044	ref	
	1	143	42.541	0.578(0.332,1.007)	
	2	154	32.645	0.206(0.103,0.412)	
≥60					
	0	79	48.619	ref	
	1	102	42.149	0.781(0.488,1.249)	
	2	61	25.207	0.58(0.327,1.032)	

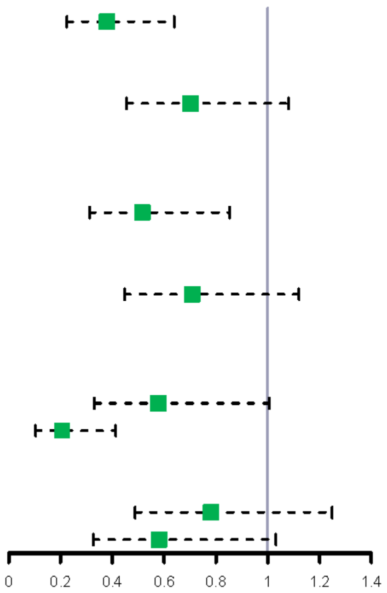

Figure S4. Subgroup interaction analysis stratified by age evaluating the associations of LCR, GNRI, and LCR_GNRI with OS in patients with GSRCC. OS, overall survival; GSRCC, gastric signet ring cell carcinoma; LCR, lymphocyte-to-C-reactive protein ratio; GNRI, Geriatric Nutritional Risk Index; LCR_GNRI, combined LCR–GNRI score.

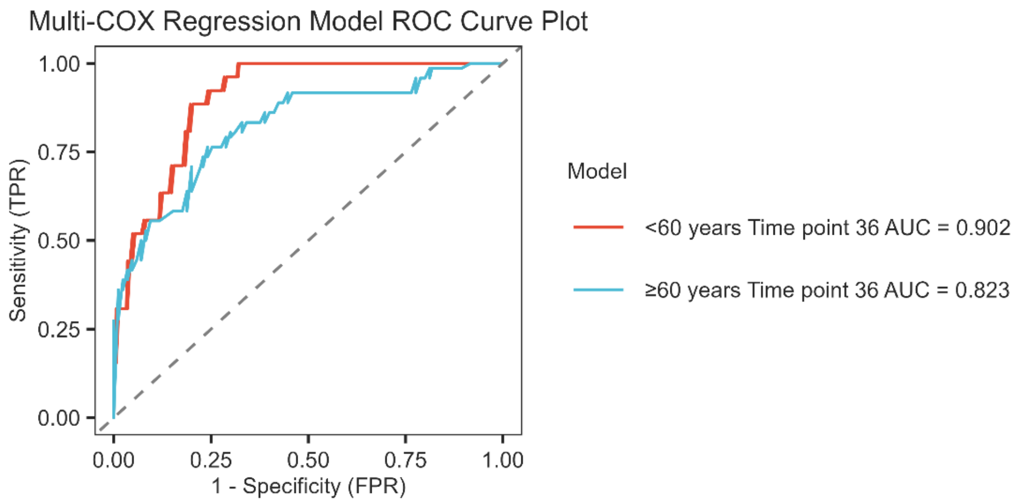

Figure S5. ROC curves comparing the predictive performance of the nomogram between patients aged <60 years and ⩾60 years in the GSRCC cohorts for 3-year OS. OS, overall survival; GSRCC, gastric signet ring cell carcinoma; ROC, receiver operating characteristic curve.

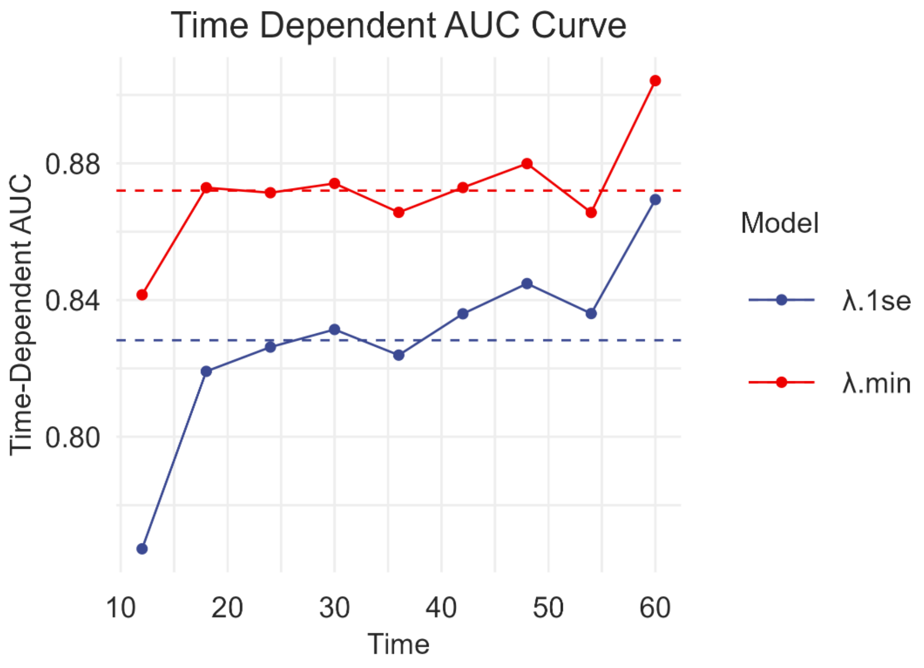

Figure S6. t-ROC curves of models with λ.min and λ.1se for prediction of OS. The horizontal axis shows postoperative survival time, and the vertical axis displays the estimated AUC. λ.min (red, with minimum cross-validation error) and λ.1se (blue, within one standard error of the minimum); t-ROC, time-dependent receiver operating characteristic curve; AUC, area under the curve; OS, overall survival.
